# Symptomatic, functional and quality of life measures of remission in 194 outpatients with schizophrenia followed naturalistically in a 6-month, non-interventional study of aripiprazole once-monthly

**DOI:** 10.1038/s41537-023-00405-5

**Published:** 2023-11-08

**Authors:** Christoph U. Correll, Andreas Brieden, Wolfgang Janetzky

**Affiliations:** 1 The Zucker Hillside Hospital, Department of Psychiatry, Northwell Health, Glen Oaks, NY USA; 2 Donald and Barbara Zucker School of Medicine at Hofstra/Northwell, Department of Psychiatry and Molecular Medicine, Hempstead, NY USA; 3 Charité Universitätsmedizin Berlin, Department of Child and Adolescent Psychiatry, Berlin, Germany; 4 Universität der Bundeswehr München, Werner-Heisenberg-Weg 39, D-85577 Neubiberg, Germany; 5 Lundbeck GmbH, Ericusspitze 2, 20457 Hamburg, Germany

**Keywords:** Schizophrenia, Psychosis

## Abstract

An important goal in the treatment of patients with schizophrenia is remission in various domains, i.e., of symptoms, psychosocial functioning and subjective well-being. We undertook a post hoc analysis of pre-stabilized outpatients with schizophrenia and complete outcome data who had been enrolled in a 6-month non-interventional study of aripiprazole once-monthly (AOM) at 75 German sites. Key outcomes were (i) symptomatic remission (cross-sectional Andreasen et al. criteria (≤mild positive and negative key symptoms on the Brief Psychiatric Rating Scale (BPRS))); (ii) functional remission (Global Assessment of Functioning (GAF) scale score >70), and (iii) subjective well-being remission (WHO-5 scale score ≥13) at week 24. Of 242 enrolled patients, 194 (80.2%) (age = 43.9 ± 15.3 years; 51.5% male, illness duration = 14.0 ± 12.0 years) with complete data were analyzed. While 61.3% of the patients achieved symptomatic remission and 76.8% achieved remission regarding subjective well-being, only 24.7% achieved psychosocial functioning remission at 6 months. Remission rates were similar for men and women and across strata of disease duration with, on average, less remission in patients with longer illness duration. Correlations of improvements on the BPRS and GAF were weak, with the weakest correlation between the BPRS depressive mood item and the GAF scale, but similarly high correlation between BPRS subscales or the BPRS depressive mood item and subjective well-being. These findings suggest that while treatment with AOM can lead to symptomatic remission and remission regarding subjective well-being, additional interventions such as psychosocial therapy or supported employment and education may be necessary to achieve functional remission.

## Introduction

Remission is an important treatment goal in schizophrenia. A consensus definition of symptomatic remission in schizophrenia and operational criteria for its assessment (also known as the Andreasen et al. criteria) were published in 2005 by the Remission in Schizophrenia Working Group^1^. The criteria encompass core symptoms of schizophrenia, as assessed by the Positive and Negative Syndrome Scale (PANSS) or Brief Psychiatric Rating Scale (BPRS), which are required to be no more than “mild” (3 points) for a duration of ≥6 months. These criteria were subsequently found to be valid, achievable and sustainable for a meaningful proportion of patients^2^. Different rates of remission are observed based on the studied patient population. While in first episode psychosis up to 81% of patients in remission have been reported^3^, the usual percentages lie between 40 and 60% in patients with chronic/multi-episode non-affective psychotic disorders^2^. Moreover, a diagnosis of schizophrenia is linked to poorer outcomes compared with other schizophrenia-spectrum disorders^2^. Poor odds of achieving remission are found in patients with male sex, younger age at illness onset, poorer premorbid adjustment and more severe baseline psychopathology^4^. Some risk factors for poor odds of remission are modifiable, which include longer duration of untreated illness, nonadherence to antipsychotics, comorbidities (especially substance use disorders), lack of early antipsychotic response and lack of improvement with non-clozapine antipsychotics, which is predictive of clozapine response^4^. Being able to achieve symptomatic remission is relevant, as patients who achieve remission are less likely to relapse than patients who do not achieve remission during assured antipsychotic treatment^5^.

Symptomatic remission is also related to the concept of recovery, which also includes functional remission with or without adequate health-related quality of life and well-being, depending on the definitions used^6–9^. However, recovery is not a well-defined concept, lacking a reliable metric. Proposed criteria are remission of symptoms plus remission in another dimension related to broader social functioning for a duration of ≥2 years^6^. Consensus definitions are also still lacking for functional remission and adequate well-being or health-related quality of life^2,10^. Although patients who achieve remission tend to have better functional and health-related quality of life outcomes, they do not necessarily achieve a state of remission in these additional dimensions^2,8^. Moreover, the relationship between health-related quality of life and illness severity is complex, with some data suggesting that patients with poor illness insight and poor cognition report better health-related quality of life, while those with better illness insight and cognition, and in particular depressive symptoms, report poorer health-related quality of life^11,12^.

Adherence to treatment is an important predictor of remission^2,13^. With each subsequent relapse, the odds of achieving remission become smaller^14,15^. For example, in a study of subjects with up to four psychotic episodes, 17% failed to remit after each episode, irrespective of which episode it was^16^. Therefore, long-term maintenance treatment and relapse prevention are crucial in terms of achieving and sustaining remission^17,18^. Antipsychotic treatment prevents relapse with a number needed to treat of 3^19^, and second-generation antipsychotics are more effective in preventing relapse than first-generation antipsychotics^20^. Furthermore, use of long-acting injectable antipsychotics (LAIs) has advantages over oral medication^21^. LAIs have been found to be more effective in preventing relapse and hospitalization^22^ while being as safe as their oral counterparts^23,24^ and offering long-term advantages in terms of reduced mortality^25^. These advantages of LAIs over oral antipsychotics are more obvious in studies that are closer to everyday clinical practice than randomized controlled trials^26^. In a recent cohort study, individuals were 67% less likely to stop medication if they were on an LAI compared to oral medication^27^. Even when patients stop antipsychotic medication, there seems to be some remaining protection against relapse after LAI use compared to oral use^28^. However, in clinical practice, LAI use is still insufficient despite its advantages^27^. A greater focus on remission, functioning and well-being is needed, which is linked to assured antipsychotic maintenance treatment to enable more effective and less interrupted psychosocial rehabilitation and reintegration.

The aim of this non-interventional study of the LAI aripiprazole once-monthly (AOM) in outpatients with schizophrenia was to assess the frequency of single and multiple concurrent dimensions of remission, as well as some basic demographic and illness characteristics as potential predictors of remission defined on the following three levels: remission of symptoms, functional remission and remission regarding subjective well-being. Based on prior literature, we hypothesized that symptomatic remission would be easier to achieve than functional remission, with less clearly predictive patterns regarding subjective well-being.

We used data from a non-interventional study in Germany for our analysis, the results of which have been reported^29,30^. Briefly, it was a multicenter, prospective, non-interventional study that included 242 patients who started treatment with AOM after their treating physician had prescribed it, and were then monitored for 6 months. Among the endpoints were psychopathology (Brief Psychiatric Rating Scale, BPRS), psychosocial functioning (Global Assessment of Functioning, GAF) and well-being (WHO-5 well-being index). The patients had been pretreated with oral aripiprazole for 9.7 months on average (±22.3) and 87.9% were regarded clinically stable by their treating physicians, with the stable condition having lasted for a mean of 5.9 months. During the study, the mean BPRS total score improved from 54.1 ± 15.6 at study start, with an improvement of −13.8 (±16.0) during 6 months. The mean GAF score at baseline was 47.0 (±13.9), and increased to 60.2 (±17.0) during 6 months. Patients reported a mean WHO-5 score of 10.6 (±5.6) at study start, and the score increased during 6 months to 15.4 (±5.5). 204 patients (84.3%) completed all scheduled visits, and 23 patients (9.5%) came for at least the first and last visits.

## Results

Of 242 enrolled patients, 194 (80.2%) (age = 43.9 ± 15.3 years; 51.5% male, illness duration = 14.0 ± 12.0 years) had complete data and were analyzed. Baseline data are presented in Table 1. In order to account for the patients not analyzed here, we compared the baseline data of our analyzed patient with those of all 242 patients (Table 1).

**Table 1:** Baseline characteristics of the patient sample in this study.

Variable	Total sample (*n* = 194)	Patients in symptomatic remission at baseline (*n* = 44)	Patients not in symptomatic remission at baseline (*n* = 150)	*p* value	Total cohort from the original study (*n* = 242)
Age, years, mean (SD)	43.9 (15.3)	43.6 (14.3)	43.9 (15.7)	0.903	43.1 (15.1)
Sex, male, *n* (%)	100 (51.5)	24 (54.5)	76 (50.7)	0.651	133 (55.0)
Illness duration, years, mean (SD)	14.0 (12.0)	12.8 (11.3)	14.3 (12.2)	0.439	13.5 (12.5)
BMI, kg/m², mean (SD)^a^	29.1 (7.0)	30.7 (5.3)	28.6 (7.4)	0.032	29.3 (6.9) (*n* = 240, FAS)
BPRS total score at baseline, mean (SD)	54.6 (15.7)	35.7 (5.9)	60.2 (13.2)	<0.001	53.4 (15.9) (*n* = 239, FAS)
GAF score at baseline, mean (SD)	47.2 (13.8)	57.0 (12.6)	44.3 (12.9)	<0.001	48.6 (14.8) (*n* = 240, FAS)
WHO-5 score at baseline, mean (SD)	10.5 (5.3)	12.7 (4.6)	9.9 (5.4)	0.001	10.7 (5.6) (*n* = 238, FAS)

*SD* standard deviation, *BMI* body mass index, *BPRS* Brief Psychiatric Rating Scale, *GAF* Global Assessment of Functioning, *WHO-5* World Health Organization-5 well-being index, *FAS* full analysis set.

^a^For one patient in remission at baseline the information on weight is not available. In this case all values are calculated for 193 patients.

### Symptomatic remission—BPRS

**Fig. 1:**
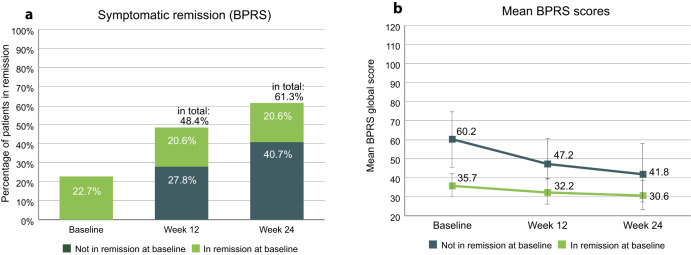
Symptomatic remission. **a** Percentages of patients in symptomatic remission as per cross-sectional Andreasen et al. criteria, determined by BPRS. Percentages refer to total population (*n* = 194). **b** Mean BPRS global scores of patients in remission or not in remission at baseline. Error bars show standard deviation. BPRS Brief Psychiatric Rating Scale.

The proportion of patients in symptomatic remission at baseline was 22.7% and rose to 61.3% at study endpoint (Fig. 1a). 79 patients (40.7%) were not in remission at baseline and achieved remission during the study. 40 patients (20.6%) remained in remission throughout the study. Mean BPRS total scores, stratified by remission status at baseline, are shown in Fig. 1b. Patients in remission at baseline had an average score of 35.7 (SD 5.9) at study start and 30.6 (SD 7.1) at endpoint, whereas patients not in remission at baseline had an average score of 60.2 (SD 13.2) at study start and 41.8 (SD 14.7) at endpoint.

**Fig. 2:**
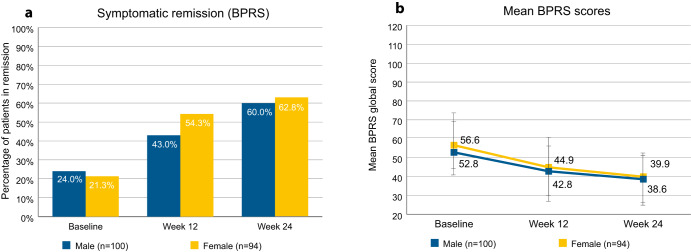
Symptomatic remission as determined by BPRS, stratified by sex. **a** Percentages of patients in symptomatic remission as per cross-sectional Andreasen et al. criteria, determined by BPRS. Percentages refer to total number of male (*n* = 100) or female patients (*n* = 94). **b** Mean BPRS global scores, stratified by sex. Error bars show standard deviation. BPRS Brief Psychiatric Rating Scale.

Stratifying remission by patient sex (Fig. 2) yielded little difference between the sexes. At baseline, 24.0% of the male and 21.3% of the female patients were in symptomatic remission. At study endpoint, this was the case in 60.0% of the male and 62.8% of the female patients. Male patients had average scores of 52.8 (SD 15.8) at baseline and 38.6 (SD 13.9) at endpoint, and female patients had average scores of 56.6 (SD 15.5) at baseline and 39.9 (SD 14.3) at endpoint.

Remission in relation to illness duration is shown in supplementary table 1. The odds of achieving remission tended to be greater for patients with shorter illness duration, with 67.9% of the patients with ≤5 years of duration achieving remission at week 24, compared to only 43.2% of the patients with an illness duration of >20 years.

### Functional remission—GAF

**Fig. 3:**
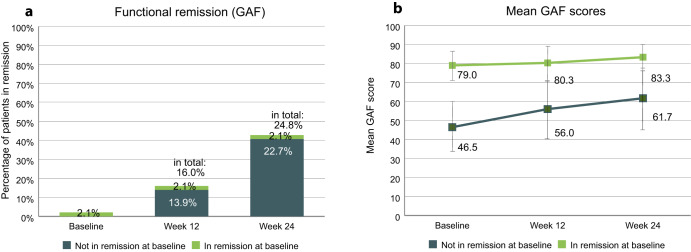
Functional remission. **a** Percentages of patients in functional remission. Patients were considered to be in remission if they had a GAF total score of >70. Percentages refer to total population (*n* = 194). **b** Mean GAF scores of patients in remission or not in remission at baseline. Error bars show standard deviation. GAF Global Assessment of Functioning.

The proportion of patients in functional remission, as determined by the GAF scale, was 2.1% at baseline and reached 24.8% at study endpoint (Fig. 3a). 44 patients (22.7%) were not in remission at baseline and achieved remission during the study. 4 patients (2.1%) remained in remission throughout the study. Mean GAF scores, stratified by remission status at baseline, are shown in Fig. 3b. Patients who were in remission at baseline had an average score of 79.0 (SD 7.3) at study start and 83.3 (SD 7.1) at study endpoint, whereas patients not in remission at baseline had an average score of 46.5 (SD 13.1) at study start and 61.7 (SD 16.3) at study endpoint.

**Fig. 4:**
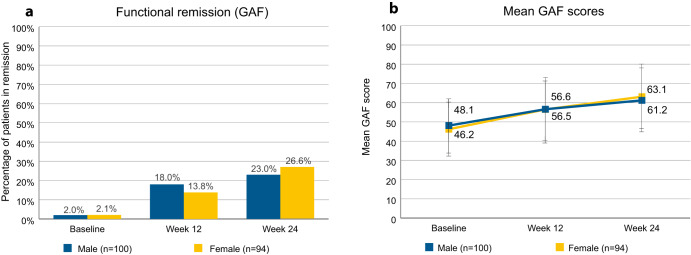
Functional remission as determined by GAF, stratified by sex. **a** Percentages of patients in functional remission. Patients were considered to be in remission if they had a GAF total score of >70. Percentages refer to total number of male (*n* = 100) or female patients (*n* = 94). **b** Mean GAF scores of patients by sex. Error bars show standard deviation. GAF Global Assessment of Functioning.

Stratifying functional remission by patient sex (Fig. 4) yielded little difference between the sexes. 2.0% of the male patients and 2.1% of the female patients were in remission at baseline, and 23.0% of the male patients and 26.6% of the female patients were in remission at study endpoint. Male patients had average scores of 48.1 (SD 13.4) at study start and 61.2 (SD 16.3) at study endpoint, whereas female patients had average scores of 46.2 (SD 14.2) at study start and 63.1 (SD 16.6) at study endpoint.

Functional remission in relation to illness duration is shown in supplementary table 2. Again, odds of achieving remission tended to be greater for patients with shorter illness duration, with 33.9% of the patients with ≤5 years of illness duration achieving functional remission at week 24, compared to only 13.6% of the patients with an illness duration of >20 years.

### Subjective well-being (WHO-5)

**Fig. 5:**
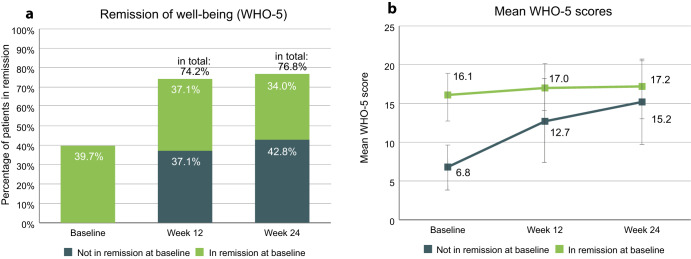
Remission of subjective well-being as determined by WHO-5. **a** Percentages of patients in remission regarding their subjective well-being. Patients scoring at least 13 points were considered in remission. Percentages refer to total population (*n* = 194). **b** WHO-5 scores of patients in remission or not in remission at baseline. Error bars show standard deviation. WHO-5 World Health Organization-5 Well-Being Index.

The proportion of patients in remission in terms of subjective well-being, as determined by the WHO-5 scale, was 39.7% at baseline and reached 76.8% at study endpoint (Fig. 5a). 83 patients (42.8%) were not in remission at baseline and achieved remission during the study. 63 patients (32.5%) remained in remission throughout the study. Mean WHO-5 scores, stratified by remission status at baseline, are shown in Fig. 5b. Patients who were in remission at baseline had an average score of 16.1 (SD 2.5) at study start and 17.2 (SD 4.1) at endpoint, whereas patients not in remission at baseline had average scores of 6.8 (SD 3.0) at study start and 15.2 (SD 5.7) at endpoint.

**Fig. 6:**
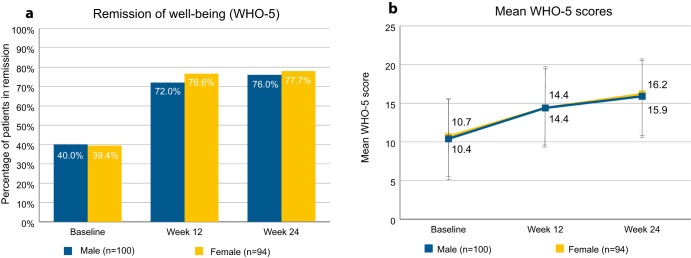
Remission of subjective well-being as determined by WHO-5, stratified by sex. **a** Percentages of patients in remission. Percentages refer to total number of male (*n* = 100) or female patients (*n* = 94). **b** WHO-5 scores of patients by sex. Error bars show standard deviation. WHO-5 World Health Organization-5 Well-Being Index.

Stratifying remission of subjective well-being by patient sex (Fig. 6) yielded little difference between the sexes. At baseline, 40.0% of the male patients and 39.4% of the female patients were in remission, and at study endpoint, this was the case for 76.0% of the male and 77.7% of the female patients. Male patients had average scores of 10.4 (SD 5.5) at baseline and 15.9 (SD 5.0) at study endpoint, and female patients had average scores of 10.7 (SD 5.2) at baseline and 16.2 (SD 5.5) at study endpoint.

Subjective well-being remission in relation to illness duration is shown in supplementary table 3. Here, remission of well-being was fairly evenly distributed across duration strata.

### Remission on multiple rating scales

**Fig. 7:**
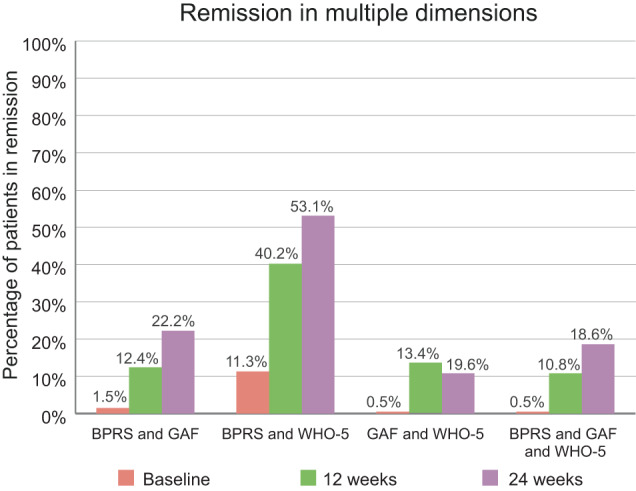
Remission in multiple dimensions, as assessed via multiple rating scales. Percentages refer to total population (*n* = 194). BPRS Brief Psychiatric Rating Scale, GAF Global Assessment of Functioning, WHO-5 World Health Organization-5 Well-Being Index.

We assessed the proportion of patients who remitted in multiple dimensions (that is, according to different scales) over time (Fig. 7). Only 22.2% of the patients achieved both symptomatic and functional remission at the end of the study, but 53.1% achieved symptomatic remission and remission of well-being. 18.6% of the patients achieved remission in all three dimensions.

### Correlations of changes in rating scales

Correlations of changes in the rating scales are presented in Table 2. The highest correlations (about 70% each) were seen for the different subscales of the BPRS with each other; the lowest correlation was between the GAF score and the BPRS depressive mood item (35.0%). The correlation of the GAF and WHO-5 scores with the BPRS subscale scores was intermediate at about 50–60%, as was the correlation of the WHO-5 score with the BPRS depressive mood item.

**Table 2:** Correlation of absolute changes in rating scales (BPRS reduction, GAF and WHO-5 increase).

	BPRS positive items	BPRS negative items	BPRS general items	BPRS general items w/o Depressive mood	BPRS item Depressive mood	GAF score	WHO-5 score
BPRS positive items		72.3%	73.1%	71.1%	59.4%	59.3%	47.3%
BPRS negative items			70.7%	69.5%	55.1%	55.8%	52.2%
BPRS general items						55.6%	54.4%
BPRS general items w/o Depressive mood					65.2%	57.3%	51.5%
BPRS item Depressive mood						35.0%	48.7%
GAF score							41.6%
WHO-5 score							

Correlations are color coded, with red highlighting minimal correlation and green highlighting maximal correlation. Correlations between BPRS general items and BPRS general items w/o Depressive mood or the BPRS item Depressive mood were not calculated, as Depressive mood is part of the BPRS general items.

For all BPRS items: absolute reduction, for GAF and WHO: absolute increase. BPRS positive items are: Conceptual disorganization, Mannerisms and posturing, Grandiosity, Hostility, Suspiciousness, Hallucinatory behavior, Unusual thought content, Excitement. BPRS negative items are: Emotional withdrawal, Motor retardation, Uncooperativeness, Blunted affect, Disorientation. BPRS general items are: Somatic concern, Anxiety, Guilt feelings, Tension, Depressive mood.

*BPRS* Brief Psychiatric Rating Scale, *GAF* Global Assessment of Functioning, *WHO-5* World Health Organization-5 Well-Being Index.

## Discussion

Symptomatic remission is an important treatment goal, paving the way for recovery. In this post hoc analysis of outpatients from a 6-month non-interventional study in Germany who were treated with AOM, we found that symptomatic remission was achieved by 61.3% of the patients in our study. Other studies found that 40–60% of patients achieve remission^2^, which is consistent with our results.

It is noteworthy that 87.9% of the original 242 patients were considered stable by their treating physicians^30^, but only 22.7% of the analyzed population was in symptomatic remission at study start. Stability was assessed at the discretion of the treating physician, so that we do not have insights as to what criteria have been employed by them to determine stability. Possible criteria may have included a stable dose of oral aripiprazole during pre-treatment, or no change in symptoms over a certain period of time. Staring AOM, as well as starting participation in a study (which may have led to better and more regular interaction with the patient), may have improved adherence to the medication, which in turn may have resulted in improvements in the outcomes studied here.

Remission of well-being was achieved by 76.8% of the patients, but functional remission by only 24.8%. The result that functional remission is less often achieved than symptomatic remission and remission of well-being was also found in a larger observational study with 2960 German outpatients^31^. There, at endpoint, 47.2% of the patients achieved symptomatic remission, 26.6% achieved functional remission, and 42.2% achieved adequate subjective well-being. A different study found more symptomatic than functional remission in first-episode patients^32^. Other studies found that symptomatic remission is associated with better functional outcomes, but not necessarily functional remission^33^. Likewise, the patients in our study experienced improvements on the GAF, as reported previously^29^, but not enough to achieve remission. It seems, therefore, that antipsychotic medication can lead to remission of symptoms and adequate well-being, but in order for most patients to regain adequate functional levels, additional interventions such as psychosocial therapy or supported employment are needed.

In general, we found no differences between the sexes regarding remission, showing that both women and men can benefit similarly from AOM treatment in terms of symptomatic remission and remission of well-being and are likewise similarly in need of additional help in order to achieve functional remission. In terms of functional remission, women tended to show slightly larger improvements during the study than men.

Looking at remission rates stratified by illness duration, we found a trend toward less remission in longer lasting disease. This finding is probably due to accumulated relapses over time. After each relapse, the likelihood of remission tends to get smaller^14–16^, which highlights the need for early assured antipsychotic treatment with an LAI, which is currently the best available measure for relapse prevention^22^.

In order to achieve recovery from schizophrenia, remission in multiple dimensions is needed^6^. Ideally, symptomatic remission should be achieved together with functional remission, so that social participation and a normal level of education and employment are possible, as well as adequate well-being and health-related quality of life. However, this goal is difficult to attain. Only 22.2% of the patients analyzed here achieved remission of both symptoms and functioning, and only 18.6% achieved remission in all three studied dimensions (symptoms, functioning and well-being).

When looking at correlation levels of improvements in different dimensions, we found the highest correlations (about 70%) among the BPRS subscales, intermediate correlations between BPRS subscales or BPRS depressive mood item and subjective well-being (about 50%), and the lowest correlation between the BPRS depressive mood item and GAF (35%). This result reflects further evidence that additional measures may be necessary to achieve good functional outcomes in patients with schizophrenia. Thus, as described before, depression is strongly correlated with subjective well-being^11^, providing a valuable treatment target in patients with schizophrenia and comorbid depression. In contrast, depression alone was poorly correlated with functioning, indicating that other factors, including global psychopathology, are more relevant for functional deficits in schizophrenia.

Limitations of our study include the fact that this was a post hoc analysis and that the number of studied patients was limited. This study inherits the limitations of the original study, which are due to its naturalistic, non-interventional design^30^. There was no control group, and possible confounding factors cannot be identified or excluded. Patients may have been inadvertently “selected” (patients who tolerated and responded to oral aripiprazole, patients who were willing to take LAI medication), and there may have been expectation bias due to the open-label design of the study. Moreover, for our analysis, we used only data from patients with complete datasets. This offers the advantage of having the same basis for all analyses done here, but reduces the number of evaluable patients from 242 in the original study to 194 taken into account here. Also, patients who drop out of a study are less likely to achieve remission^2^, therefore our sample is likely to be enriched with patients who would be more likely to achieve remission.

Furthermore, the Remission in Schizophrenia Working Group defined remission as a mild or less level of key symptoms, maintained for 6 months^1^. We omitted the duration criterion here, using only cross-sectional criteria. Since the study duration was only 6 months, we would have been unable to record changes over time when applying the duration criterion. Also, the BPRS does not include two key negative symptoms, namely social withdrawal and lack of spontaneity, making the use of the BPRS a “softer” remission criterion than use of the Positive and Negative Syndrome Scale (PANSS)^2^.

Another limitation is the use of the GAF to assess functioning, since its score depends on either functioning or symptom severity, whichever is worse at the time of rating^34,35^. It would be better to use a scale that is focused on functioning, such as for example the Social and Occupational Functioning Scale (SOFAS) or the Functional Remission of General Schizophrenia Scale (FROGS)^36^. For our study, we chose a GAF cut-off at >70 points to define remission, because this level reflects at most mild impairment in social, occupational or school functioning^37^, and we felt that this definition corresponded well to the Andreasen et al. criteria for symptomatic remission. Other groups have used a GAF score of >60^38,39^ or >80^40,41^ as a cut-off for remission, reflecting the lack of a consensus definition.

Our remission of well-being criterion was a WHO-5 score of at least 13. We chose this value because it has been reported that values below 12.5 indicate possible depression^42^.

Despite the limitations, however, we were able to identify an important aspect of schizophrenia treatment here, namely that treatment with a long-acting injectable antipsychotic may lead to remission of symptoms and well-being, but remission of functioning is less likely, suggesting that additional interventions are needed that can benefit from the achieved symptomatic remission.

## Conclusion

In this non-interventional study, we found in a sample of adult outpatients with schizophrenia that the fraction of patients who achieved symptomatic remission and adequate well-being was much larger than the fraction who achieved functional remission. It seems that for most patients, antipsychotic medication is not sufficient to achieve functional remission and that additional interventions, such as psychosocial therapy or supported employment, are needed to restore psychosocial functioning.

## Methods

This was a post hoc analysis of data from a 6-month, multicenter, prospective, non-interventional study in Germany that included 242 ambulatory patients with schizophrenia treated at 75 centers who were switched to aripiprazole once-monthly and monitored for 24 weeks^29,30^. Data from 194 patients with complete datasets (80.2%) of originally 242 patients were used for this post hoc analysis.

Our goal was to analyze remission rates over time in three different domains (symptomatic, functional and subjective well-being), and the relationships between these different types of remission. We chose to only analyze patients with complete data, so that we would be able to analyze composite endpoints while using the same data as a basis for all analyses.

### Definitions of remission

Symptomatic remission was defined according to the cross-sectional Andreasen et al. criteria^1^, omitting the time criterion of ≥6 months duration. We considered patients to be in remission when the remission criteria-relevant items of the Brief Psychiatric Rating Scale (BPRS) were at most mild (≤3). These remission criteria-relevant items were Grandiosity, Suspiciousness, Unusual thought content, Hallucinatory behavior, Conceptual disorganization, Mannerisms/posturing and Blunted affect.

Functional remission was defined as a Global Assessment of Functioning (GAF) score of >70, reflecting at most mild symptoms or slight impairment in social, work or school functioning^37^.

Remission in terms of subjective well-being was defined as a WHO-5 well-being index score of ≥13^42^.

### Data analysis

We calculated percentages of remitted patients, as well as rating scale score means and standard deviations.

Furthermore, we calculated correlations of absolute changes on rating scales. For this analysis, we divided the BPRS into its original positive, negative and general psychopathology subscale. BPRS positive items are: Conceptual disorganization, Mannerisms and posturing, Grandiosity, Hostility, Suspiciousness, Hallucinatory behavior, Unusual thought content, Excitement. BPRS negative items are: Emotional withdrawal, Motor retardation, Uncooperativeness, Blunted affect, Disorientation. BPRS general items are: Somatic concern, Anxiety, Guilt feelings, Tension, Depressive mood. In addition, the BPRS Depressive mood item was analyzed separately due to literature linking depression to subjective well-being and health-related quality of life in schizophrenia^11^. In order to reflect clinical improvements on each scale, we used absolute reduction for the BPRS subscales and absolute increase for GAF and WHO-5.

### Supplementary information

Measures of remission, stratified by duration of disease

## Data Availability

The data this paper is based on is available from the authors upon reasonable request.
