# Supplementary material for: Symptomatic, functional and quality of life measures of remission in 194 outpatients with schizophrenia followed naturalistically in a 6-month, non-interventional study of aripiprazole once-monthly
Source: Schizophrenia (Heidelb). 2023 Nov 8;9(1):80. doi: 10.1038/s41537-023-00405-5 (PMC10630463; doi:10.1038/s41537-023-00405-5)
Supplement: Supplementary file 1 — Measures of remission, stratified by duration of disease [file 41537_2023_405_MOESM1_ESM.pdf]

Symptomatic, Functional and Quality of Life Measures of Remission in 194 Outpatients with Schizophrenia Followed Naturalistically in a 6-month, Non-Interventional Study of Aripiprazole Once-Monthly

Christoph U. Correll, Andreas Brieden, Wolfgang Janetzky

Supplement

Supplementary table 1: Symptomatic remission as determined by BPRS, stratified by duration of disease.

Duration (years)	Number of patients	Remitted at baseline, n (%)	Mean BPRS global score (SD)	Remitted at week 12, n (%)	Mean BPRS global score (SD)	Remitted at week 24, n (%)	Mean BPRS global score (SD)
0-5	56	11 (19.6)	54.4 (14.3)	32 (57.1)	42.8 (13.5)	38 (67.9)	38.0 (12.7)
6-10	37	13 (35.1)	50.6 (18.7)	23 (62.2)	42.4 (17.7)	25 (67.6)	37.0 (15.4)
11-15	36	8 (22.2)	56.3 (16.1)	14 (38.9)	43.4 (15.3)	24 (66.7)	38.8 (14.2)
16-20	21	5 (23.8)	55.6 (16.0)	12 (57.1)	43.0 (17.4)	13 (61.9)	41.5 (16.6)
>20	44	7 (15.9)	56.6 (14.5)	13 (29.5)	47.1 (14.8)	19 (43.2)	42.0 (13.4)

BPRS, Brief Psychiatric Rating Scale; SD, standard deviation.

Supplementary table 2: Functional remission as determined by GAF, stratified by duration of disease.

Duration (years)	Number of patients	Remitted at baseline, n (%)	Mean GAF score (SD)	Remitted at week 12, n (%)	Mean GAF score (SD)	Remitted at week 24, n (%)	Mean GAF score (SD)
0-5	56	1 (1.8)	47.0 (13.2)	12 (21.4)	58.7 (17.7)	19 (33.9)	64.3 (17.3)
6-10	37	1 (2.7)	50.0 (12.6)	8 (21.6)	59.5 (17.4)	11 (29.7)	64.0 (17.0)
11-15	36	1 (2.8)	50.9 (12.9)	5 (13.9)	61.1 (12.1)	8 (22.2)	66.7 (12.9)
16-20	21	0 (0.0)	45.2 (13.4)	2 (9.5)	53.7 (12.8)	4 (19.0)	57.4 (16.9)
>20	44	1 (2.3)	43.0 (15.6)	4 (9.1)	49.6 (17.6)	6 (13.6)	56.3 (15.7)

SD, standard deviation; GAF, Global Assessment of Functioning.

Supplementary table 3: Remission of subjective well-being as determined by WHO-5, stratified by duration of disease.

Duration (years)	Number of patients	Remitted at baseline, n (%)	Mean WHO-5 score (SD)	Remitted at week 12, n (%)	Mean WHO-5 score (SD)	Remitted at week 24, n (%)	Mean WHO-5 score (SD)
0-5	56	19 (33.9)	9.3 (5.0)	41 (73.2)	14.0 (5.1)	43 (76.8)	16.1 (5.2)
6-10	37	12 (32.4)	10.4 (5.2)	27 (73.0)	13.9 (5.4)	30 (81.1)	16.5 (4.2)
11-15	36	14 (38.9)	10.2 (5.6)	29 (80.6)	14.5 (5.2)	28 (77.8)	15.8 (6.0)
16-20	21	11 (52.4)	11.4 (4.8)	16 (76.2)	15.0 (5.0)	15 (71.4)	14.9 (5.4)
>20	44	21 (47.7)	11.9 (5.8)	31 (70.5)	14.9 (4.8)	33 (75.0)	16.3 (5.4)

SD, standard deviation; WHO-5, World Health Organization-5 Well-Being Index.
